# Spontaneous Decoding of the Timing and Content of Human Object Perception from Cortical Surface Recordings Reveals Complementary Information in the Event-Related Potential and Broadband Spectral Change

**DOI:** 10.1371/journal.pcbi.1004660

**Published:** 2016-01-28

**Authors:** Kai J. Miller, Gerwin Schalk, Dora Hermes, Jeffrey G. Ojemann, Rajesh P. N. Rao

**Affiliations:** 1 Departments of Neurosurgery, Stanford University, Stanford, California, United States of America; 2 NASA—Johnson Space Center, Houston, Texas, United States of America; 3 Program in Neurobiology and Behavior, University of Washington, Seattle, Washington, United States of America; 4 National Center for Adaptive Neurotechnologies, Wadsworth Center, New York State Department of Health, Albany, New York, United States of America; 5 Psychology, Stanford University, Stanford, California, United States of America; 6 Department of Neurological Surgery, University of Washington, Seattle, Washington, United States of America; 7 Center for Sensorimotor Neural Engineering, University of Washington, Seattle, Washington, United States of America; 8 Computer Science and Engineering, University of Washington, Seattle, Washington, United States of America; Indiana University, UNITED STATES

## Abstract

The link between object perception and neural activity in visual cortical areas is a problem of fundamental importance in neuroscience. Here we show that electrical potentials from the ventral temporal cortical surface in humans contain sufficient information for spontaneous and near-instantaneous identification of a subject’s perceptual state. Electrocorticographic (ECoG) arrays were placed on the subtemporal cortical surface of seven epilepsy patients. Grayscale images of faces and houses were displayed rapidly in random sequence. We developed a template projection approach to decode the continuous ECoG data stream spontaneously, predicting the occurrence, timing and type of visual stimulus. In this setting, we evaluated the independent and joint use of two well-studied features of brain signals, broadband changes in the frequency power spectrum of the potential and deflections in the raw potential trace (event-related potential; ERP). Our ability to predict both the timing of stimulus onset and the type of image was best when we used a combination of both the broadband response and ERP, suggesting that they capture different and complementary aspects of the subject’s perceptual state. Specifically, we were able to predict the timing and type of 96% of all stimuli, with less than 5% false positive rate and a ~20ms error in timing.

## Author Summary

We describe a new technique for decoding perception from electrical potentials measured from the human brain surface. All previous attempts have focused on the identification of classes of stimuli or behavior where the timing of experimental parameters is known or pre- designated. However, real world experience is spontaneous, and to this end we describe an experiment predicting the occurrence, timing, and types of visual stimuli perceived by human subjects from the continuous brain signal. In this experiment, human patients with electrodes implanted on the underside of the temporal lobe were shown pictures of faces and houses in rapid sequence. We developed a novel template-projection method for analyzing the electrical potentials, where, for the first time, broadband spectral changes and raw potential changes could be contrasted as well as combined. Our analyses revealed that they carry different physiological information, and, when used together, allow for unprecedented accuracy and precision in decoding human perception.

## Introduction

How does a two-dimensional pattern of pixels measured by our retina get transformed into the percept of a friend’s face or a famous landmark? It is known that the ventral temporal cortex represents different classes of complex visual stimuli within distinct regions. For example, category-selective areas have been established unambiguously at scale of several millimeters using functional imaging and macroscale field potentials [1–4]. Similar results have also been demonstrated at the single-unit level in epileptic human patients [5] and non-human primates [6]. More recently, high frequency electrocorticographic (ECoG) changes from these same ventral temporal regions have been shown to increase while viewing images of faces, places, and other objects [7–10]. However, rather than reflecting a discrete range of frequencies, >40Hz ECoG changes have been shown to instead be a reflection of broadband fluctuations across the entire frequency domain [11,12], and these broadband changes show robust increases across ventral temporal cortex during object perception [13].

**Fig 1:**
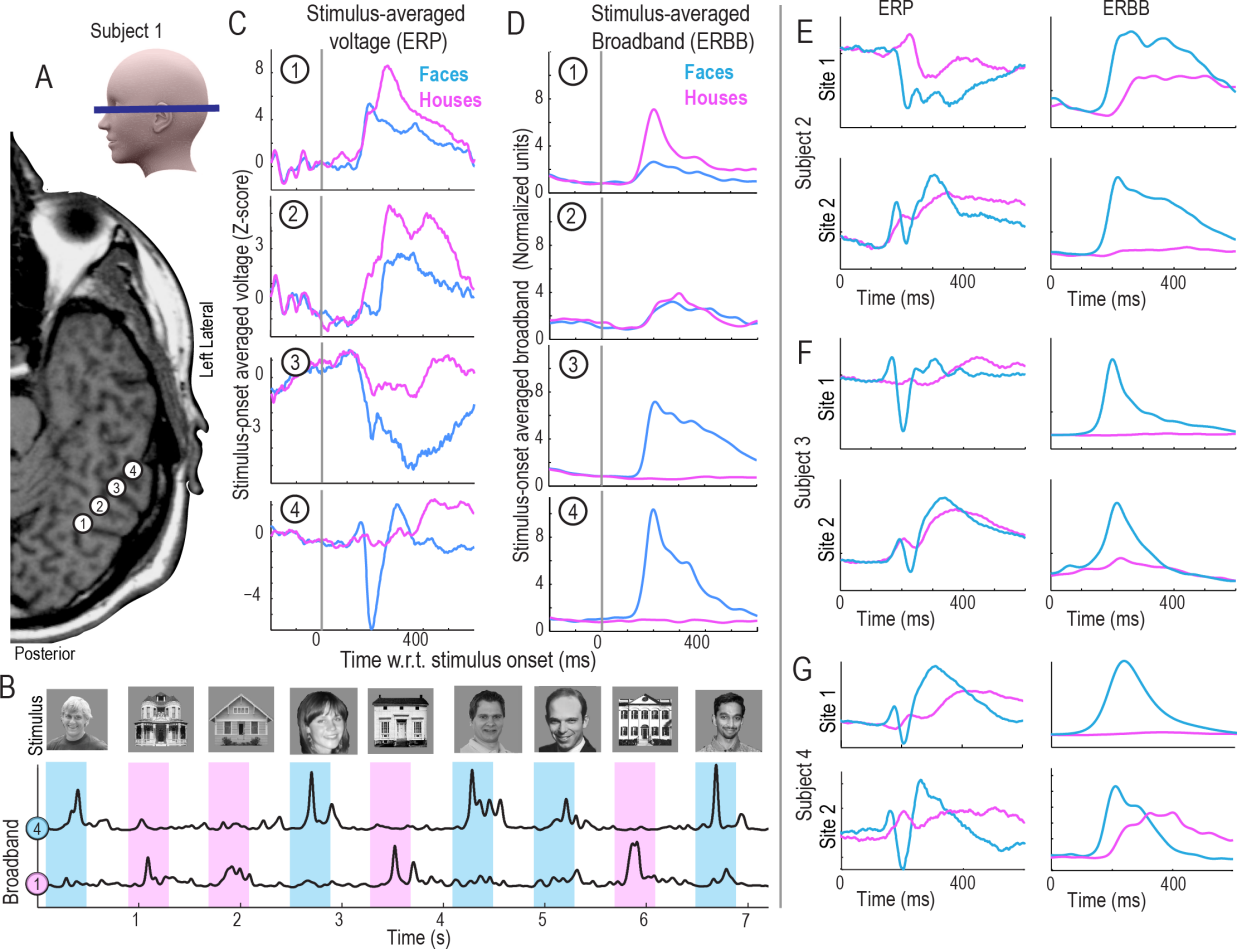
The basic face and house discrimination task, and the polymorphic nature of the electrophysiologic response. **(A)** Subdural electrocorticographic (ECoG) electrode strips were placed through burrholes in the skull onto ventral temporal brain surface. 4 adjacent sites are shown for subject 1. **(B)** Simple luminance- and contrast-matched grayscale faces and houses that were displayed in random order for 400ms each, with 400ms blank gray screen inter-stimulus interval between each picture. Subjects were asked to report a simple target (an upside-down house, which was rejected from analyses). From the raw potential, the time course of broadband spectral change was extracted from each brain site (here sites 1&4 from (A)). Blue = faces; pink = houses. **(C)** The averaged raw potential (ERP) following face (blue) and house (pink) stimuli for the 4 sites in (A). **(D)** The averaged broadband power following different stimuli (ERBB–a reflection of averaged neuronal firing rate), from sites 1–4 in (A). **(E-G)** ERBB and ERP for 2 sites over fusiform gyrus in subjects 2–4. Note that the responses are highly polymorphic for the event-related potentials, and that there are ERP face-selective sites that do not have the classic N200 shape. As seen for site 2 in Subject 4, the classic N200, when present, does not guarantee face-selectivity in the ERBB.

Object-category specific responses in inferotemporal cortex were initially identified using event-related potentials (ERPs) in ECoG [14,15] or functional magnetic resonance imaging (fMRI) [1–4] although little spatial overlap was found between the ERP and the fMRI response [16]. In contrast, increases in high-frequency broadband power in cortical surface potentials recorded using ECoG matched well with the category-specific fMRI responses in the inferior temporal cortex [17,18]. The ERP and broadband signals show distinct, and partially overlapping, responses to faces [13,19] (Fig 1), but it is unclear whether the information content is itself distinct between the two. While both the ERP and the raw ECoG potential have previously been used to classify object categories [20–22], these studies required knowledge about the time of stimulus onset, rather than determining them spontaneously. Furthermore, the ability of the algorithms to establish object category from neural data was well below that of human performance (both in terms of accuracy and temporal fidelity).

A significant methodological obstacle to this type of macroscale physiology has been the difficulty interpreting heterogeneity in response morphologies. As illustrated in Fig 1, face-selective ERPs may have wide structural variation, with “peaks” and “troughs” that are very different in shape, latency, and duration, even when measured from brain sites separated by only 1cm. It remains unclear what the ERP shape actually corresponds to. Furthermore, methodology has not previously been developed to naively place morphologically-diverse ERPs in a common feature space. In contrast, broadband spectral changes in the ECoG signal have been shown to correlate with neuronal firing rate [23,24], although it has been unclear how ERPs relate to this, or what the best way to attempt such a comparison is [19]. Our work begins by describing a template-projection technique, where templates of averaged raw potentials (ERPs) and broadband changes (ERBB) from a training period are projected into the data from a testing period. This places ERP and ERBB features from different brain sites into a common feature space, where they can be directly compared with one another, and used together for decoding brain function.

To date, decoding of perceptual content has relied upon designated information about external stimuli, where the frequency of occurance and precise timing are known to the decoder. We propose that in addition to identifying the perceptual content (e.g. image type), decoding of the brain state should evolve to spontaneously identify whether a perceptual event has happened from the datastream, and, if so, predict the timing as accurately as possible. We denote this practice as “spontaneous decoding”.

Here we show that the ECoG signal contains sufficient information to allow near-instantaneous identification of object categories with an accuracy comparable to that of human behavioral performance. Our experiments measured ECoG recordings from several inferior temporal visual areas simultaneously while subjects viewed randomly interleaved images of faces or houses. We achieved the best results by combining broadband changes with raw potential changes (rather than with either independently), using a template projection approach. This shows that the two types of signals capture complementary aspects of the physiology reflecting a human subject’s perceptual state. With this combination, we were able to predict 96% of all stimuli correctly as face, house, or neither, with only ~20 ms error in timing.

## Methods

### Ethics statement

All patients participated in a purely voluntary manner, after providing informed written consent, under experimental protocols approved by the Institutional Review Board of the University of Washington (#12193). All patient data was anonymized according to IRB protocol, in accordance with HIPAA mandate. A portion of this data appears in a different context in [13]. All data and analyses are publically available at http://purl.stanford.edu/xd109qh3109.

### Subjects and recordings

All 7 subjects in the study were epileptic patients (S1 Table) at Harborview Hospital in Seattle, WA. Subdural grids and strips of platinum electrodes (Ad-Tech, Racine, WI) were clinically placed over frontal, parietal, temporal, and occipital cortex for extended clinical monitoring and localization of seizure foci. Lateral frontoparietal electrode grids were discarded from analysis, and only strip electrodes were further considered. The electrodes had 4 mm diameter (2.3 mm exposed), 1 cm inter-electrode distance, and were embedded in silastic. Electrode locations relative to gyral surface anatomy were determined by projection of the post-implant CT to the pre-operative axial T1 using normalized mutual information in SPM, and the CTMR package, with Freesurfer-extracted cortical surface mesh reconstructions [25–28]. When the MRI or CT was of insufficient quality, hybrid techniques were used [29].

Experiments were performed at the bedside, using Synamps2 amplifiers (Neuroscan, El Paso, TX) in parallel with clinical recording. Stimuli were presented with a monitor at the bedside using the general-purpose BCI2000 stimulus and acquisition program [30]. The electrocorticographic potentials were measured with respect to a scalp reference and ground, subjected to an instrument-imposed bandpass filter from 0.15 to 200 Hz, and sampled at 1000 Hz.

To reduce common artifacts, the potential, $V_{n}^{0}\left(t\right)$, measured at time *t* in each electrode *n*, was re-referenced with respect to the common average of all *N* electrodes, $V_{n}\left(t\right)=V_{n}^{0}\left(t\right)-\frac{1}{N}\sum_{i=1}^{N}V_{i}^{0}\left(t\right)$. Electrodes with significant artifact or epileptiform activity were rejected prior to common averaging. There was no rejection of epochs of time within the data. Ambient line noise was rejected by notch filtering between 58–62 Hz using a 3^rd^-order Butterworth filter [31].

### Face-House discrimination task

Subjects performed a basic face and house stimulus discrimination task. They were presented with grayscale pictures of faces and houses (luminance- and contrast-matched) that were displayed in random order for 400ms each, with 400ms blank screen inter-stimulus interval (ISI) between the pictures. The 10cm-wide pictures were displayed at ~1m from the patients while they were seated at the bedside (Fig 1). There were 3 experimental runs with each patient, with 50 house pictures and 50 face pictures in each run (for a total of 300 stimuli). In order to maintain fixation on the stimuli, patients were asked to verbally report a simple target (an upside-down house), which appeared once during each run (1/100 stimuli). There were few errors in reporting the upside-down target house in any run (approximately 2–3 across all 21 experimental runs).

### Power spectral analysis, and decoupling the dynamic power spectrum to obtain the timecourse of broadband spectral change (fully detailed in the Supplemental material, S1 Text and S2 Text)

Following previously described methodology [11,32,33], we perform discrete estimates of the windowed power spectrum, as well as a time-frequency approximation of the dynamic power spectrum from *V*_*n*_(*t*). We then perform a “decoupling process” to identify underlying motifs in power-spectral change, isolating the timecourse of broadband spectral change, *B*_*n*_(*t*). This process was originally described and illustrated in full detail for ECoG recordings from motor cortex [11], and later illustrated specifically for this face-house context [12]. Broadband changes have been shown to robustly characterize the magnitude and latency of cortical dynamics from ventral temporal cortex, in single trials, during this face and house viewing experiment [13]. Generically, the broadband power time course is meant to function as a time-varying estimate of changes in a multiplicative factor of the population firing rate [11,24].

### Decoding

#### Cross-validation

Prior to further analysis, the data were divided into thirds temporally (e.g. divided into experimental runs). Subsequent analyses were then performed in a 3-fold fashion. In each cross-fold, two thirds (two runs) of the data were assigned to a **“training” set**, and the remaining third was assigned to a **“testing” set** (In bold throughout for emphasis). In this way, all data could be used for both testing as well as training, but never at the same time (to maximize use without “double-dipping”, which is simultaneously testing and training on the same data). However, the spectral decoupling process was performed only once, across all data, rather than cross-folded (the decoupling process is ignorant of class-labels, or timepoint selection).

#### Template projection technique

*Stimulus triggered averaged raw potential and broadband template*: In each electrode *n*, stimulus-triggered averages of the training data were obtained for the common-averaged electric potential for the face (*S* → *F*) and house (*S* → *H*) stimuli independently ($\tau_{k_{s}}$ denotes the *k* ^*th*^ of *N*_*S*_ total instances of stimulus type *S* in the training set):

$${\langle V_{n}\left(t'\right)\rangle }_{S}^{0}=\frac{1}{N_{S}}\sum_{k_{s}=1}^{N_{S}}V_{n}\left(\tau_{k_{s}}+t'\right).$$

This quantity is only calculated on the peri-stimulus interval −199 < *t*' ≤ 400 ms (where *t*' denotes time with respect to stimulus start). It is then re-centered by subtracting the average potential peri-stimulus baseline on the interval −199 < *t*' ≤ 50, (50ms post-stimulus is chosen to correspond with ERP and broadband ECoG latency to primary visual cortex [33,34]) to obtain 〈*V*_*n*_(*t*')〉_*S*_:

$${\langle V_{n}\left(t'\right)\rangle }_{S}={\langle V_{n}\left(t'\right)\rangle }_{S}^{0}-\frac{1}{250}\sum_{t"=-199}^{50}{\langle V_{n}\left(t"\right)\rangle }_{S}^{0}$$

We perform the same averaging over the **training data** for the broadband signal to obtain 〈*B*_*n*_(*t*')〉_*S*_. Examples of these response templates, 〈*V*_*n*_(*t*')〉_*S*_ and 〈*B*_*n*_(*t*')〉_*S*_ are illustrated throughout the manuscript.

#### Projection of templates into pre-defined times of stimuli onset (illustrated in Fig 2)

〈*V*_*n*_(*t*')〉_*S*_ and 〈*B*_*n*_(*t*')〉_*S*_ were generated from the **training period**.

**Fig 2:**
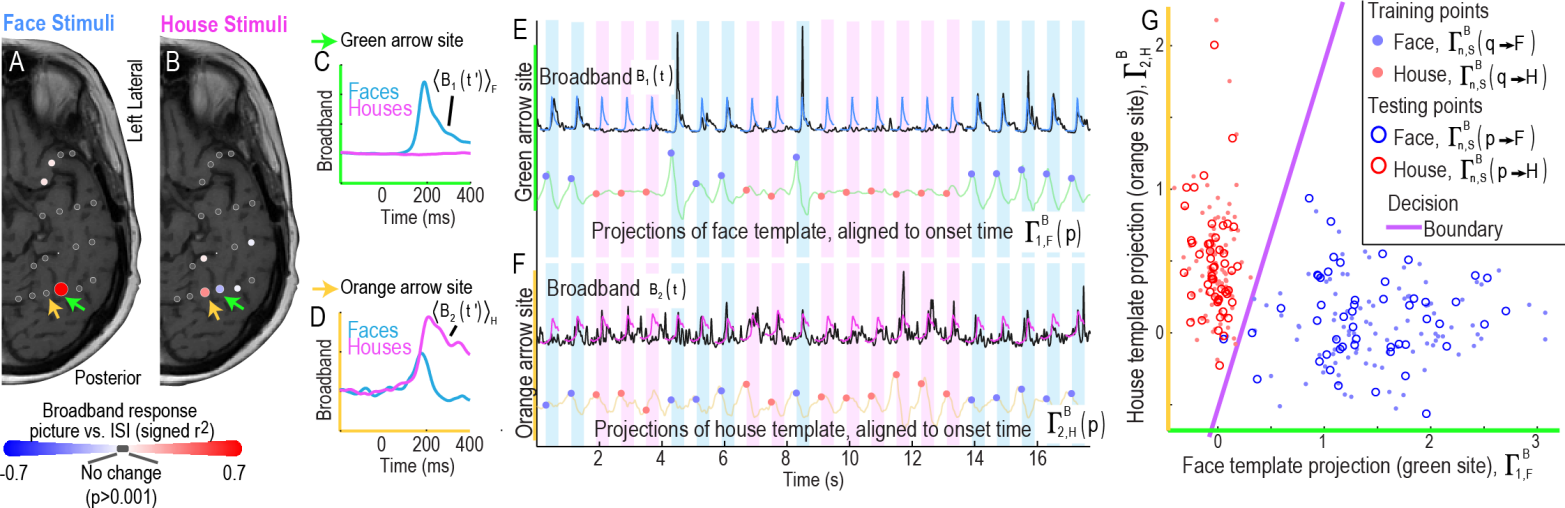
Decoding the stimulus class in single trials when the onset of a stimulus is known, subject 3. **(A)** Squared cross-correlation values at each electrode. **Training feature points** were obtained by back-projecting the event triggered broadband, 〈*B*_*n*_(*t*')〉_*F*_ (see Methods), into the training data and comparing projected face, $\Gamma_{n,F}^{B}\left(q\to F\right)$, and inter-stimulus interval (ISI), $\Gamma_{n,F}^{B}\left(q\to o\right)$, points. These $r_{n}^{2}$ values are scaled by color, and plotted on an axial MRI slice with scaling shown in the colored bar beneath. The electrodes meeting acceptance criteria $r_{n}^{2}>0.05$ were selected as features for classification for the face template. **(B)** As in (A), but for house stimuli from the training period. **(C)** Event-triggered broadband templates from the training period for face, 〈*B*_1_(*t*')〉_*F*_, and house, 〈*B*_1_(*t*')〉_*H*_ stimuli, from the electrode noted with a green arrow in (A-B). **(D)** As in (C), but from the electrode noted with an orange arrow. (**E)** Projection of event-triggered face template from (C) into **testing data**: The top black trace shows a portion of the broadband time course from the electrode noted with a green arrow, during the testing period, *B*_1_(*t*). The 〈*B*_1_(*t*')〉_*F*_ face template is shown in light blue at each stimulus time, irrespective of class, at event testing times *τ*_*p*_. The result of projecting the face template 〈*B*_1_(*t*')〉_*F*_ to *B*_1_(*t*) is shown in the green background trace, $\Gamma_{1,F}^{B}\left(t\right)$, with testing points at defined face stimulus times, $\Gamma_{1,F}^{B}\left(p\to F\right)$, shown with blue circles, and defined house stimulus times, $\Gamma_{1,F}^{B}\left(p\to H\right)$, shown with red circles. **(F)** As with (E), but for the orange-arrow electrode, *B*_2_(*t*), and using the house template from (D), 〈*B*_2_(*t*')〉_*H*_. **(G)** The subspace $\Gamma_{1,F}^{B}$ vs $\Gamma_{2,H}^{B}$, is used to illustrate discrete classification approach. Here the back-projected training points $\Gamma_{n,S}^{B}\left(q\right)$ are shown with dots (blue for *q* → *F* and red for *q* → *H*), along with the testing feature points $\Gamma_{n,S}^{B}\left(p\right)$ shown with circles. One may see that a simple decision line (purple) in this subspace would result in only 1 error.

**Training feature points** were obtained by **back-projecting** 〈*V*_*n*_(*t*')〉_*S*_ and 〈*B*_*n*_(*t*')〉_*S*_ into the **training period** to obtain sets $\Gamma_{n,S}^{V}\left(q\right)$ and $\Gamma_{n,S}^{B}\left(q\right)$ for each event *q* at time *τ*_*q*_:

$\Gamma_{n,S}^{V}\left(q\right)=\sum_{t'=-199}^{400}{\langle V_{n}\left(t'\right)\rangle }_{S}\left(V_{n}\left(\tau_{q}+t'\right)-\bar{V_{n}^{b}\left(\tau_{q}\right)}\right)$, where $\bar{V_{n}^{b}\left(\tau_{q}\right)}$ represents an “instantaneous” baseline surrounding time *τ*_*q*_: $\bar{V_{n}^{b}\left(\tau_{q}\right)}=\sum_{t=-199}^{50}V_{n}\left(t+\tau_{q}\right)$. $\Gamma_{n,S}^{B}\left(q\right)$ were obtained in the same fashion. The training event types *q* were face picture stimulus onset (*q* → *F*), house picture stimulus onset (*q* → *H*), or randomly chosen points during the inter-stimulus interval (ISI, *q* → *o*), with 4 during each ISI period, at least 100ms from stimulus offset/onset and 50ms from one another.

**Fig 3:**
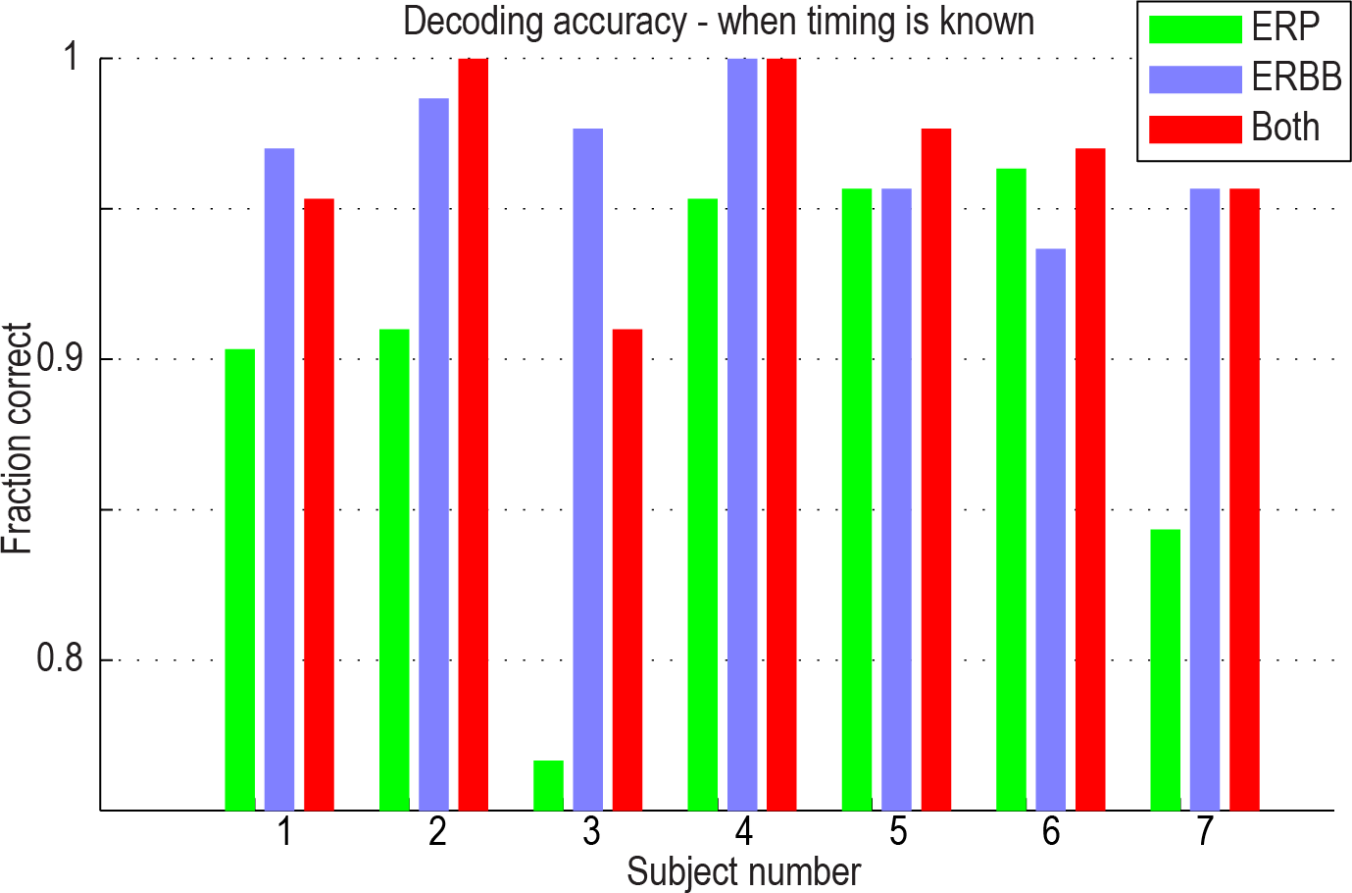
Classification accuracy when the onset of a stimulus is known, using ERP, ERBB, or both template types. In some subjects, 100% accuracy was reached. All accuracies were above 90% when both raw potential and broadband templates were used.

**Testing feature points for discrete classification,** $\Gamma_{n,S}^{V}\left(p\right)$ and $\Gamma_{n,S}^{B}\left(p\right)$, were similarly obtained by **forward-projecting** 〈*V*_*n*_(*t*')〉_*S*_ and 〈*B*_*n*_(*t*')〉_*S*_ into the **testing period** for pre-defined times of face or house picture stimuli onset events, *p*, at times *τ*_*p*_. These results are illustrated in Fig 3.

#### Projection of templates into continuous data stream (illustrated in Figs 4–6)

To quantify how well the averaged raw potential 〈*V*_*n*_(*t*')〉_*S*_ is represented in the voltage time series of the testing data at time *t*, it is directly forward-projected onto the continuous time series at each millisecond: $\Gamma_{n,S}^{V}\left(t\right)=\sum_{t'=-199}^{400}{\langle V_{n}\left(t'\right)\rangle }_{S}\left(V_{n}\left(t+t'\right)-\bar{V_{n}^{\tau }\left(t\right)}\right)$, where $\bar{V_{n}^{\tau }\left(t\right)}$ was obtained in the same fashion as above. The same projection is performed for the broadband template 〈*B*_*n*_(*t*')〉_*S*_, to obtain $\Gamma_{n,S}^{B}\left(t\right)$.

**Fig 4:**
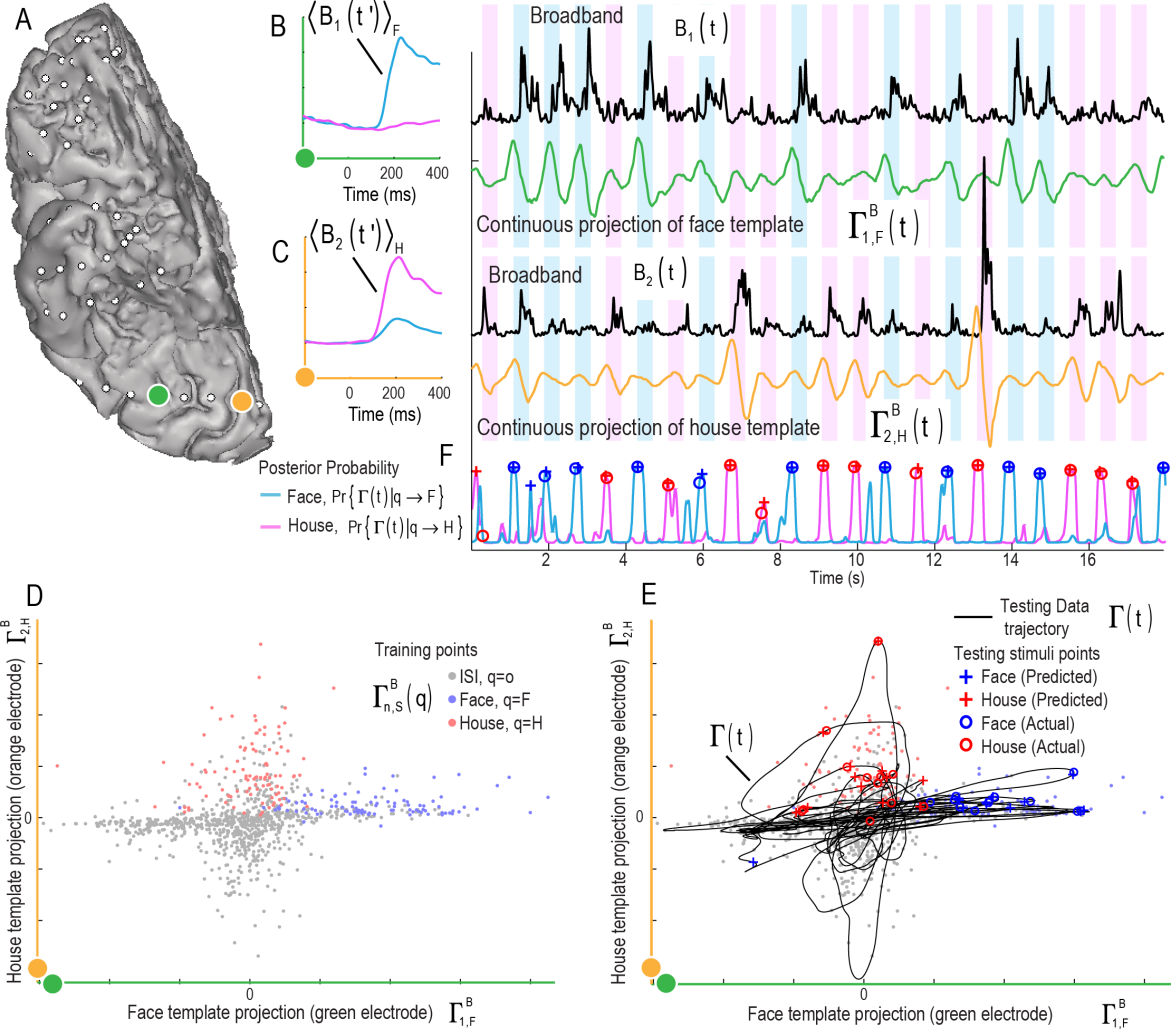
Decoding stimulus class and onset time from a continuous data stream in single trials: Illustration of two electrodes and the continuous classifier using 2 broadband features (subject 2). **(A)** Two cortical sites (3 cm from one another) on the fusiform (green) and lingual (orange) gyri are examined. **(B)** Broadband **training** templates from the green electrode for faces (blue, 〈*B*_1_(*t*')〉_*F*_) and houses (pink, 〈*B*_1_(*t*')〉_*H*_) are shown on the axes to the left. **Testing** time course of green electrode broadband spectral change, *B*_1_(*t*), is shown to the right in black, with the projection of the face template 〈*B*_1_(*t*')〉_*F*_ into *B*_1_(*t*) to produce $\Gamma_{1,F}^{B}\left(t\right)$, shown in the green trace beneath. **(C)** As in B, but for the orange electrode site, using projections of a house template 〈*B*_2_(*t*')〉_*H*_ to produce $\Gamma_{2,H}^{B}\left(t\right)$. **(D)** The classification feature subspace is defined by back-projection of the templates on the left in (B-C), to obtain **training** points $\Gamma_{n,S}^{B}\left(q\right)$ for face, house, and ISI events at **training** times *τ*_*q*_ shown. **(E)** In order to illustrate the multi-dimensional trajectory of the brain state that emerges when different channels and features are brought into a common space, the 2D trace of $\Gamma_{1,F}^{B}\left(t\right)$ from the green electrode (B) versus $\Gamma_{2,H}^{B}\left(t\right)$ from the orange electrode (C), are shown in black in the same subspace as D. The predicted onsets for face (blue) and house (red) stimuli are shown as plus symbols while actual onsets are shown as open circles. Note that the classifier was applied to the entire broadband feature space, not just this 2D subspace. **(F)** The trajectory of the face onset posterior probability from the classifier Pr{Γ(*t*)|*q* → *F*} (blue) is shown alongside Pr{Γ(*t*)|*q* → *H*} (pink), with predicted (plus symbols) and actual (open circles) times shown.

**Fig 5:**
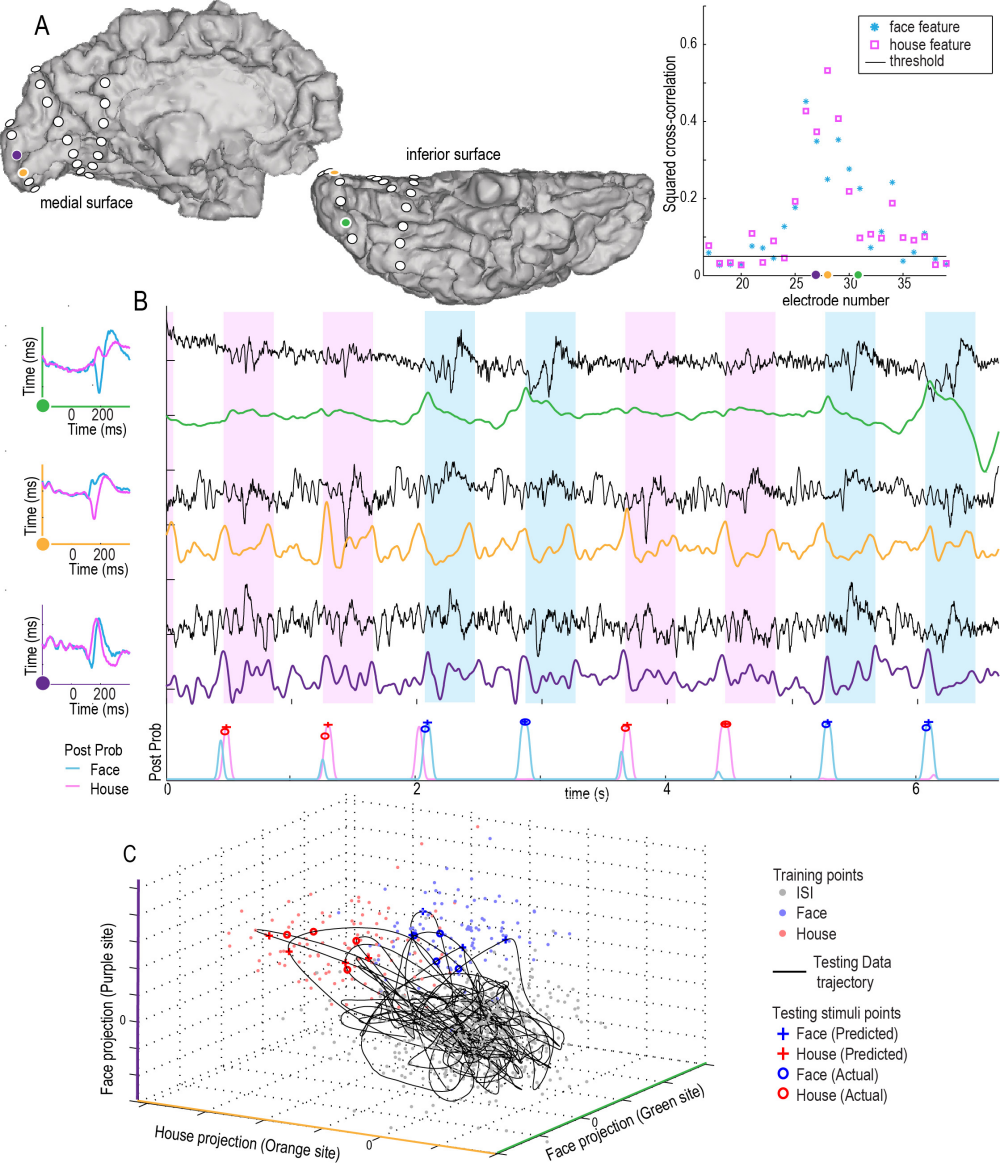
Decoding the single trial stimulus class and onset time from a continuous data stream using ERP: Illustration of three electrodes and the continuous classifier using 3 ERP-templates-to-voltage-timeseries projections (subject 4). **(A)** Three cortical sites are shown for illustration (purple, orange, and green). The axes on the right show the $r_{n,F}^{2}$ (blue asterisk, r^2^ of faces-vs-ISI) and $r_{n,H}^{2}$ (pink box, r^2^ of houses-vs-ISI) of ERP-voltage **training projections** show that these purple/orange/green sites are highly selective for faces or houses during the **training period** (from the 1^st^ cross-fold). Features falling below the black line were not used for decoding. **(B)** Averaged face and house ERPs, 〈*V*_*n*_(*t*')〉_*F*_ & 〈*V*_*n*_(*t*')〉_*H*_, from each site are shown on the left axes. These are projected into the raw voltage traces from the **testing period** (*V*_*n*_(*t*), black) to obtain continuous projection weight traces ($\Gamma_{n,F/H}^{V}$; green–face projection from green electrode, orange–house projection from orange electrode, and purple–face projection purple electrode). These traces are fed into a feature space and classified continuously to obtain posterior probability of a face, Pr{Γ(*t*)|*q* → *F*} (blue), or house stimulus, Pr{Γ(*t*)|*q* → *H*} (pink) (bottom plot). **(C)** A 3-dimensional subspace (from the sites in A and B) is illustrated, with training points from the training period shown with dots, and the subspace trajectory of the brain state, Γ(*t*), shown with a black line. Predicted and actual timing and type of stimulus are shown along this trajectory.

**Fig 6:**
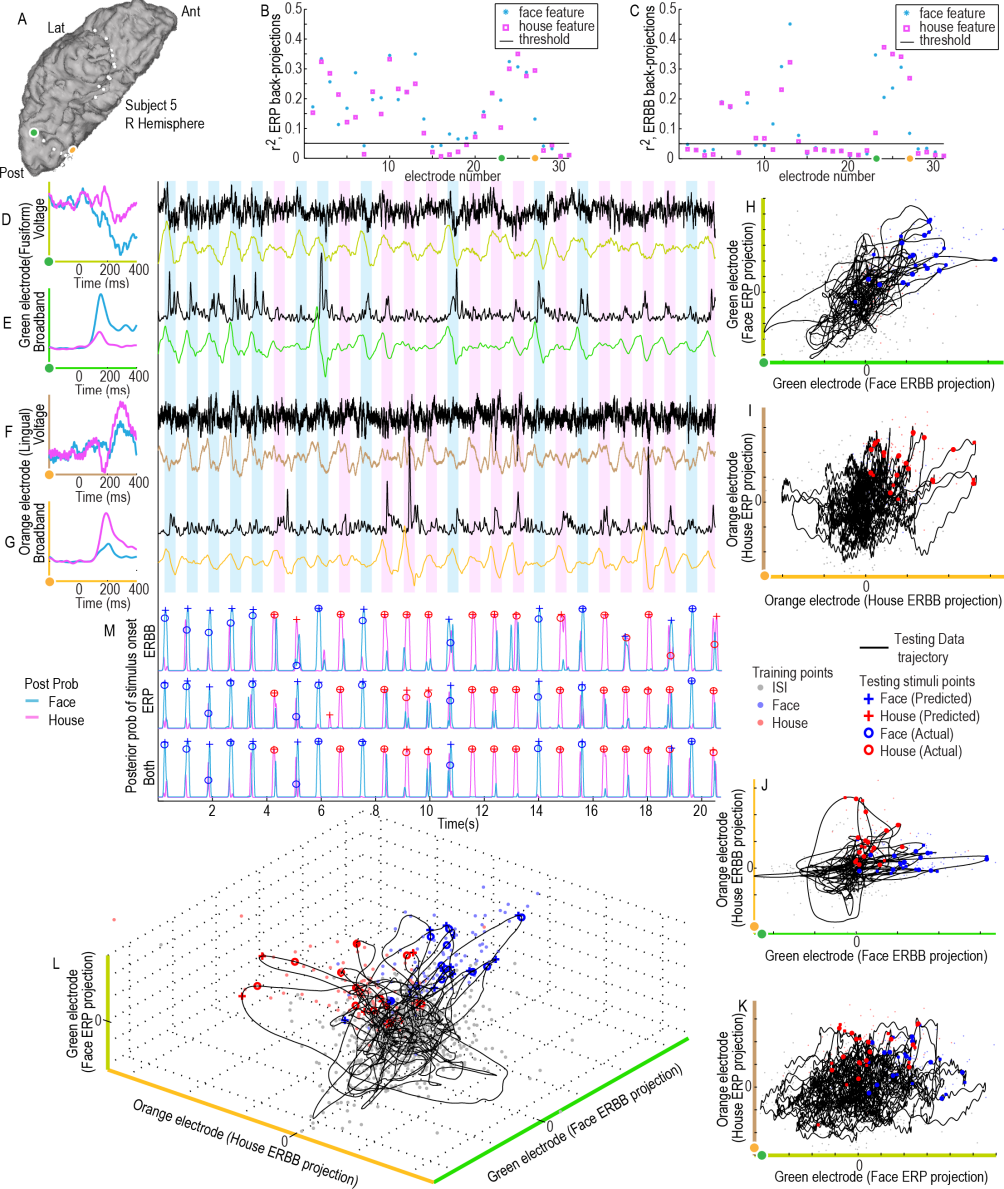
A combined ERBB-broadband and ERP-voltage projection feature space for classification (subject 5). **(A)** Two cortical sites (orange and green dots) are examined. **(B)** The axes show the $r_{n,F}^{2}$ (blue asterisk) and $r_{n,H}^{2}$ (pink box) for **ERP-voltage training** projections show that these orange and green sites are highly selective during the training period (from first cross-fold). **(C)** $r_{n}^{2}$ for **ERBB-Broadband training** projections. **(D)** Averaged face and house ERP templates from the green site are shown on the left axes (olive green). The face-ERP templates are projected into the raw voltage trace (black) to obtain continuous a projection weight trace (olive green trace). **(E)** As in (D), but for ERBB-broadband templates in the green electrode site (neon green). **(F&G)** As in (D&E), except for the orange electrode site in (A), using house ERP (brown) and ERBB (burned orange) templates. **(H)** Green electrode, face ERP vs ERBB subspace projections. **(I)** Orange electrode house ERP vs ERBB subspace projections. **(J)** ERBB projection subspace (orange-electrode house-template projection vs green-electrode face-template projection). **(K)** As in (J), for ERP projection subspace. **(L)** A 3-d subspace projection (features from D,E,G). **(M)** Posterior probability of a face, Pr{Γ_*m*_(*t*)|*q* → *F*} (blue), or house stimulus, Pr{Γ_*m*_(*t*)|*q* → *F*} (pink), having been presented (where *m* → ERP, ERBB or both features for the projection space).

#### Generation of a projection feature space

The full feature space for classification, consisting of the union of projections of the stimulus triggered average raw potentials (*V*) or broadband (*B*) across all electrodes (*n*), for faces (*F*) and houses (*H*) independently, is the combination of $\Gamma_{n,F}^{V}$, $\Gamma_{n,H}^{V}$, $\Gamma_{n,F}^{B}$, and $\Gamma_{n,H}^{B}$. For notational brevity, we can combine the notation to denote each feature as Γ_*m*_, where *m* represents a unique combination of electrode *n*, *V* or *B*, and *F* or *H*. Many of these features will not be particularly informative about when and how the brain is processing these visual stimuli, and reduce classification in the setting of a limited number of training measurements [35]. Therefore, features were individually downselected by independently assessing their squared cross-correlation between events of each stimulus type (e.g. face or house) and events drawn from the ISI during the training period, and rejecting those which fell beneath a pre-defined threshold $r_{m}^{2}<0.05$. For example, for projections of the face event-related feature, Γ_*n*,*F*_ (*V* / *B* label dropped here) we can denote the average of face stimuli as $r_{n,F}^{2}=\frac{{\left(\bar{\Gamma_{n,F}\left(q=F\right)}-\bar{\Gamma_{n,F}\left(q=o\right)}\right)}^{2}}{\sigma_{n,Fo}^{2}}\frac{N_{F}\;*\;N_{o}}{N_{Fo}^{2}}$, where *σ*_*n*,*Fo*_ is the standard deviation of the joint distribution for face and ISI events Γ_*n*,*F*_(*q* = *F*,*o*), *N*_*F*_ is the number of face events, *N*_*o*_ is the number of ISI events, and *N*_*Fo*_ = *N*_*F*_ + *N*_*o*_. In this study, we consider feature spaces consisting of projections of all types (e.g. ERP and ERBB together), and also selectively assess *B*(*t*) and *V*(*t*) independently. Example feature (sub)spaces are illustrated in Figs 2G and 4D and 4E and 5C and 6H–6L.

#### Classifier type and relation to feature space

We begin with the feature set of training points (*q*, drawn from only the training period), Γ_*m*_(*q*), where each *m* is a dimension in the feature space, and represents a particular combination of electrode, broadband or raw potential time series, and face or house template. For the sake of simplicity, Fisher linear discriminant analysis (LDA) was used for classification [36]. This characterizes the full distribution and the training period sub-distributions Γ_*m*_(*q* → *F*), Γ_*m*_(*q* → *H*), Γ_*m*_(*q* → *o*), by their means and covariances only (i.e., as if they are normally distributed). LDA assumes that the covariances of the sub-distributions are the same. Given these training distributions, data from the testing set can be assigned a posterior probability of belonging to each distribution. While we used simple LDA, one could, in principle, apply more sophisticated kernel-based or non-linear methods. Our choice of LDA was meant to simplify interpretation of our approach, which is centered on the generation of “projection feature spaces”, and provide a clear demonstration of how one may decode a continuous datastream spontaneously, rather than exploring the library of existing machine learning and classifier techniques, which is deferred to future study.

#### Classification of discrete events with known onset time (Fig 3)

We began with the case where we identify the timing of testing visual stimuli, and attempt to classify whether a face or a house picture was shown. Only the face and house training point distributions (e.g. Γ_*m*_(*q* → *F*) and Γ_*m*_(*q* → *H*)) were used to train the classifier for this discrete case. For each testing point, *p*, the assigned class was whichever posterior probability Pr{Γ(*p*)|*q* → *F*}, or Pr{Γ(*p*)|*q* → *H*}, was higher.

#### Spontaneous decoding of the continuous datastream (Fig 7)

For the prediction of type and timing of visual stimulus from continuous signal, we trained the classifier using the face (Γ_*m*_(*q* → *F*)), house (Γ_*m*_(*q* → *H*)), and ISI (Γ_*m*_(*q* → *o*)), training point distributions. Then, the LDA posterior probability that a face or house stimulus has been shown at any point in time can be measured from the testing data at each millisecond *t* as Pr{Γ(*t*)|*q* → *F*} or Pr{Γ(*t*)|*q* → *H*}. We then smooth each of these posterior probabilities with a σ = 80ms Gaussian filter, for well-behaved estimation of local maxima. From this, we assign predicted times for stimuli onset as follows: The posterior probability must be a local maximum, with value >0.51. There must be at least 320 ms between any point and the nearest assigned point (of either stimulus type–the larger posterior probability ‘wins’). A guess is considered correct if it lies within 160ms of an event. The probability of the null case, Pr{Γ(*t*)|*q* → *o*} = 1 − Pr{Γ(*t*)|*q* → *F*} − Pr{Γ(*t*)|*q* → *H*}, is >0.50 at all other times, signifying that a picture has not just been shown.

**Fig 7:**
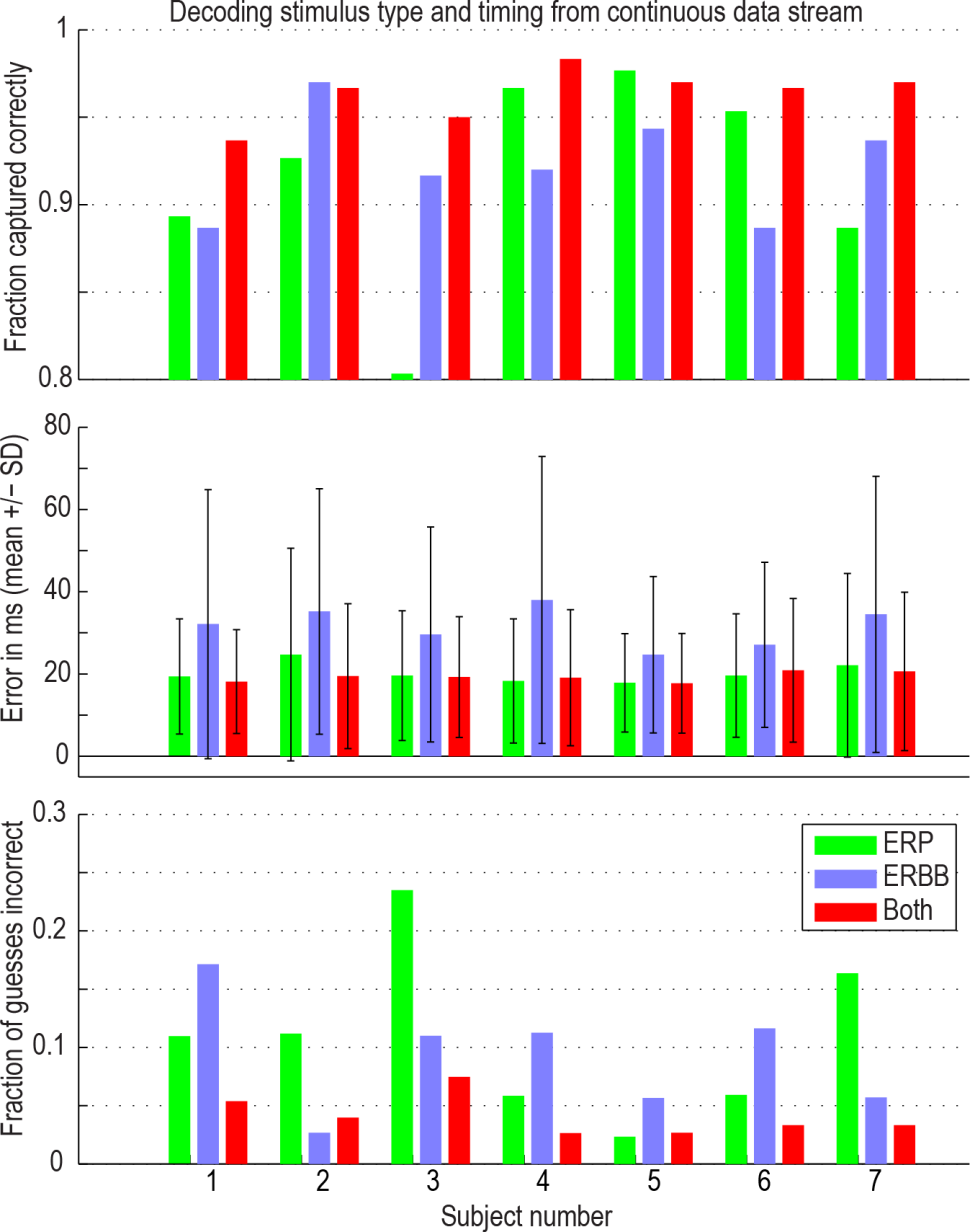
Classification accuracy for decoding stimulus class and onset in a continuous data stream. When both features were used (red bars), approximately 96% of all stimuli were captured correctly in every subject, with 15–20 ms error. An average of 4% of predictions using both features were incorrect (i.e., predicted stimuli at the wrong time, or as the wrong class). One should not confuse the fraction of guesses incorrect with the fraction of stimuli that were not captured (the bars on the top and bottom axes do not sum to 1)–it is a coincidence that also 4% of stimuli were missed.

While no information was given about the frequency of the stimuli, it was assumed that visual events were neuronally and behaviorally separable and a minimum difference of 320 ms was used. We picked 320ms as the “collision time” because we expect the neuronal response to take approximately that long [37], it makes for a random hit rate of 20% (e.g. 2.5 guesses per 800ms stimulus-to-stimulus interval, with 2 stimulus classes), and it roughly correlates with the mean broadband latencies-to-peak across single trials in these brain areas, which were found in other studies to be 269±52ms for face-selective ventral temporal sites and 299±66ms for house-selective sites [13]. This threshold for the classifier is also based in the following known aspects of the time scales of neuronal responses and face perception. First, when visual stimuli are shown in rapid order, it becomes impossible to visually distinguish each stimulus at specific rates for different stimulus classes [38,39]. For face perception this behavioral rate lies around 5–10Hz [40]. At faster rates, backward masking and temporal integration become issues. Second, the duration of a neuronal response in higher order visual areas is around 300 ms [41]. When stimuli are presented at faster rates than 300 ms each, neuronal responses from these brain areas would be expected start overlapping. Supporting information (S1 Fig) empirically shows that this choice of 320ms does not inform the classifier about frequency of stimuli shown.

In the case of spontaneous decoding of the continuous timeseries, if one were to make random guesses for events at the maximum permissible temporal density of guesses (using the rules we picked in the methodology), each guess would have a 20% chance of being correct, and 50% of stimuli would be deemed “captured”, with an 80% false positive rate, and an average temporal error of 80ms. Instead, 96% of stimuli (300 per subject) were captured, with a 4% false positive rate, and an average temporal error of 20ms.

When examining timecourses of the projections (Γ_*n*_(*t*)), as well as the resulting posterior probabilities (Pr{Γ(*t*)|*q*}), it is important to keep in mind that the templates (〈*V*_*n*_(*t*')〉_*S*_ and 〈*B*_*n*_(*t*')〉_*S*_) contain temporal information up to ~400ms later (illustrated in Figs 2–6). The local maximum of the posterior probability is the assumed to be roughly the time at ***which the templates align*** with the average response in such a way that the average response would be at the time of stimulus presentation. The portion of the signal that contributes the most to the cross-correlation is likely to be in the 150–350ms following the timepoint, *t* (based upon visual inspection of the templates and raw timecourses in Figs 1–6, as well as measured latencies in [13]).

## Results

ECoG signals were measured in seven subjects from electrodes implanted on the inferior temporal visual areas for the purpose of epilepsy monitoring. Subjects were presented with pictures of faces and houses (similar to those in Fig 1). We attempted to spontaneously identify the timing of face and house visual stimuli.

### Signal features for decoding: Event-related broadband (ERBB) and event-related potential (ERP)

To test whether the ERBB and ERP provide useful information to decode whether, when and which class of stimulus was presented, we extracted the ERBB and ERP for all electrodes. Some electrodes show a classical face-specific N200 response [13–15]. Other electrodes show face-specific ERPs with very different shapes (Fig 1).

### Decoding the stimulus class in single trials when the onset of a stimulus is known

We first investigated whether the stimulus class could be decoded in single trials when the onset of the stimulus is given. We calculated template ERBB and ERP responses from training data, which consisted of 2/3 of the recorded data (two experimental runs). The test data (for the classifier) consisted of the other 1/3 (the remaining experimental run; i.e., 3-fold cross validation, or “leave-one-run-out” cross-validation). Fig 2 shows examples of the template ERBB responses for a face- and a house-specific site. Even in a two-dimensional subspace of the full feature space, a simple line serves as a good classification boundary between the two classes of stimuli (Fig 2G).

Using either the ERP or the ERBB feature, stimuli could be robustly and reliably categorized in all cases. The average prediction accuracy using the ERBB alone was 97% across all 7 subjects, while using the ERP alone, it was 90% (Fig 3). Using a combination of the two features, 97% of stimuli could accurately be classified as face or house. It is important to note that, in subjects 1 and 3, the addition of the ERP feature actually resulted in a decrease in classification accuracy, when compared with the ERBB alone, and subject 7 shows no change. This is because of what is known as the “bias-variance tradeoff” [42,43]. For a finite number of datapoints in a training set, the inclusion of features with higher amounts of noise (ERP features in this case) can hurt overall classification. The classifier overfits noise in the mediocre features (ERP), at the expense of a tight fit to high-yield (lower noise) features (e.g. ERBB), while simultaneously expanding the size of the feature space.

### Spontaneous decoding of stimulus class and onset from a continuous cortical data stream

Figs 2 and 3 demonstrate that our analyses can accurately determine the stimulus class when given the timing of stimulus presentation. However, this type of decoding has been employed before in other experimental settings, albeit with less accuracy [20–22]. The more interesting technical question is: Can one spontaneously determine both the class and the onset of the stimuli from a continuous stream of ECoG signal features?

Our approach to the continuous decoding problem is illustrated in Figs 4–6, where template responses from a training period were applied to a period of testing data. The result of plotting the projection timeseries trajectory in a 2-dimensional subspace, Γ^*B*^(*t*), can be seen alongside training points $\Gamma_{n,S}^{B}\left(q\right)$ in Fig 4. Even in this 2-dimensional subspace projection, the furthest excursions of Γ^*B*^(*t*) into the face or house training clouds, $\Gamma_{n,S}^{B}\left(q\right)$, correlate with the times of predicted stimulus onset. Fig 5 shows an example similar to that in Fig 4, but for the ERP feature. Fig 6 shows an example of the synthesis between ERP and ERBB features when used together.

A combination between ERP and ERBB projections can be used to predict the onset timing and class of stimuli more accurately than either independently. The spontaneous classification of onset time and stimulus class was robust: 92% of stimuli were captured using the ERBB, 92% when using the ERP, and 96% of all stimuli were captured spontaneously when using a combination of both ERP and ERBB (Fig 7, top row). Furthermore, timing of stimulus onset could be predicted with approximately 20ms error when the ERP or a combination between the ERP and ERBB was used (Fig 7, middle row). The portion of incorrect predictions (e.g. false positive rate) was smallest (4%) when we used a combination of both the ERP and ERBB (i.e., predicted stimuli occured at >160ms from stimulus onset, or as the wrong class; Fig 7, bottom row).

In order to evaluate whether using both features together (ERP and ERBB) was significantly better than either independently, the labels of mean values (ERP vs ERBB vs ERP+ERBB) were randomly reshuffled (within each subject) 10^4^ times to obtain a surrogate distribution of difference in means averaged across all subjects. The 96% of events captured using both features was significantly greater than the 92% when using either independently (p = 0.0015). The timing error for correct predictions was not significantly different for both features (19ms) vs ERP (20ms, p = 0.17), but was significantly better than ERBB alone (32ms, p<0.0001). The false positive rate using both features (0.04) was significantly less than either independently (ERP 0.11; ERBB 0.09; p = 0.0012). The fact that the overall best prediction performance was reached by a combination of ERBB and ERP suggests that these two cortical features convey complementary information about a subject’s perceptual state.

Note that our 20ms estimate of the temporal fidelity of the signals may actually be an underestimate. There may be instrumentation temporal error introduced due to frame-jitter on the refresh rate of the amplifiers, sample jitter during alignment to the stimulus, and/or the granularity of sample block size of the signals imported to BCI2000 program [30]. Furthermore, there are known variations in the magnitude and timing broadband responses that are related to semantic properties (such as novelty [13]), that are disregarded in this manuscript.

We designate this technique as “Spontaneous decoding” of the ECoG datastream. Our technique processes the data, without foreknowledge of the frequency of external stimuli, nor their timing, nor their content. It then produces predictions about the occurrence, timing, and content of external stimuli, based upon a simple set of internal rules. “Spontaneous” is defined as [44]: “performed or occurring as a result of a sudden inner impulse or inclination and without premeditation or external stimulus”, and so we feel that this term is the most specific way to describe this analysis approach. While “endogenous” or “intrinsic” decoding might also have been chosen, since these are used to describe internal brain states (which is an aspect of we are actually decoding), we chose not to use them–we feel that these terms convey assumptions about the role of the temporal lobe which have yet to be proven.

## Discussion

In human experience, environmental stimuli arrive continuously, producing a sequentially evolving perceptual state. It has remained unknown whether the brain surface electrical potential has sufficient spatiotemporal fidelity to capture this dynamically changing perceptual state. Our results demonstrate that a sparse sample of the cortical surface potential contains sufficient information to reliably predict *whether* and *when* a particular stimulus occurred, with approximately the fidelity of conscious perception. It has also remained unknown whether the mesoscale neurophysiologies of event-related potentials and broadband spectral changes reflect the same information.

Previous studies aimed at decoding perception have all pre-defined the onset time of each stimulus [6,20–22,45,46]. In the first-stage of our analysis, we performed this type of classification using pre-defined onset time, with 97% accuracy (Figs 2 and 3). Similar prior studies attained representative peak accuracies of 72% with MEG/fMRI [22], 89% with EEG [20], and 94% with MEG [21]. However, real-world perception rarely occurs at pre-defined times, and approaches to decoding perceptual experience should be extracted spontaneously from continuous cortical recordings.

We have developed a technique to do just this, applying a novel template projection technique that enabled us to capture some aspects of the neural response that have previously been difficult or impossible to capture. First, the ERP in face-selective regions in the fusiform gyrus is classically associated with a negative peak at ~200ms (“N200”). Our data show that the actual shape of face-selective fusiform ERPs can vary widely, even at fusiform sites 1 cm from one another (Fig 1). The template projection technique captures these diverse response patterns, allowing them to be exploited for classification of perceptual state. Second, broadband responses show variability in the pattern of response in every individual trial. The template projection method relies on a superposition of the single trial characteristic shape and a probability density function for modeling different shapes, offering a robust prediction of perceptual state in spite of the variability across single trials. Examination of the features separately demonstrated that broadband changes are more robust and reliable reflections of perceptual content than raw-voltage changes, but that projection of ERP into raw voltage changes produces sharper temporal precision. Together, these two measures complement one another, providing independent information that results in more accurate and temporally precise prediction of the perceptual state than either measure on its’ own.

Our decoding fidelity approaches that of conscious thought, correctly capturing 96% of all stimuli from a sparsely-sampled stream of cortical potentials. The missed 4% (as well as the <5% false positive rate) approaches what might be expected for rates of inattention by hospital patients viewing multiple stimuli each second (note that random guessing at the maximum rate in this spontaneous decoding would result in a 20% chance of each guess being correct, and 50% of stimuli deemed “captured”, with an 80% false positive rate). A temporal precision of ~20ms (Fig 7, middle row) is of the same order as the post-retinal temporal granularity of the visual system [47]. These ECoG measurements show that some electrodes in early visual cortex already display some stimulus-selective responses (e.g., Fig 5, purple site). This agrees with observations that fast eye movements can be made just based upon the Fourier spectrum of the images of different classes [48], and that people saccade towards a scene containing an animal or face within 140 ms [49,50]. By demonstrating that object categories can be decoded from a continuous image stream with accuracies matching expected human behavior (e.g. attentional lapses expected at a rate of approximately 5% in a task of this type [51]), our study lays the groundwork for capturing human perceptual states in a natural environment.

Although we applied this template-projection technique to prediction, the framework may be used in a wide variety of experimental settings. ERPs from adjacent cortical regions may be highly polymorphic, complicating cross-comparison of timing and magnitude effects. In this projection space, however, trial-to-trial ERP variations from different cortical sites may be compared directly, opening a new family of analyses that might be applied to cognitive settings, where image content and context are experimentally manipulated on single trials. Similarly, one might optimize the differential strengths of each feature, such as broadband for magnitude of response and ERP for timing of response, comparing these to stimulus properties to learn about subtleties of functional specialization in each brain region.

An important feature of this template projection approach is that it provides a robust, continuous, measure that is a summary statistic for how well the brain state at every point in time reflects the expected response (e.g. as if a perceptual event or action had occurred at that time–note that the shape of the expected physiological response, however idiosyncratic, is built into the method). This could be extremely useful in settings where the cortical dynamics and latency differ by region, yet a global behavior of a distributed visual [52], auditory [53,54], motor [55], or other network must be characterized. In emerging work, this technique is implemented in a different way, to generate broadband ECoG templates from a low-noise localizer task, and apply them to a visual discrimination task at the perceptual threshold, quantifying single trial variation in cortical physiology (neuronal response magnitude and timing) [56].

Our results beg the question: What is the underlying neural basis for the increased accuracies obtained by combining ERPs with broadband activity? A direct connection between neuronal population firing rate and broadband ECoG spectral change has been established with experimental and modeling work [11,23,24]. Each clinical ECoG electrode averages over approximately 5x10^6^ neurons in the cortex beneath. Careful experimentation has shown that the broadband changes follow a power law in the power spectral density, implying that it reflects *asynchronous* spiking elements in the underlying population of neurons. The broadband measure may be loosely thought of as a real-time summation of this population’s firing raster (i.e., intrinsically averaged across the population of neurons). Increases in spike transmission within neurons in the population add in quadrature (e.g., proportional to the square root of the number of spikes), appearing as a “speeding up” of a random walk in the electrical potential time series, *are difficult to see when looking at the raw potential*, but apparent as broadband, P∼1fχ power-law, changes when inspecting in the frequency domain [24]. Recent work has shown that, in these data, the broadband timing is subtle enough to capture variational effects at the order of ~50ms due to context-dependent processing, such as sequential novelty [13].

Synchronized inputs, by contrast, add linearly and *can be easily seen in the raw tracing of the electrical potential*. Even if the synchronization is relatively weak, averaging across the neural population augments the synchronized portion, while the other aspects, such as broadband spectral change, are relatively diminished. Event-locked inputs, from subcortical nuclei, or other cortical regions, can trigger a synchronized physiologic cascade, evident at the macroscale as an ERP. It remains unclear whether the polyphasic ERP is a result of interplay between coordinated excitatory pyramidal neuron depolarization followed by interneuronal lateral inhibition, or whether it results from synaptic integration followed by characteristic depolarization and repolarization of cortical laminar dipoles [57]. The polymorphic nature of different ERPs from adjacent cortical regions may (perhaps) then relate to different pyramidal neuron morphologies, different milieus of neuronal subtypes, or different laminar organization; our projection technique unfolds these polymorphic ERPs into a common space for comparison. In this light, the improved decoding accuracy may be the result of multi-location timing information conveyed by ERP during the initial feed-forward wave of neural activation [58], complemented by the broadband response reflecting subsequent local recurrent and longer-range cortico-cortical processing of the visual stimulus.

## Supporting Information

S1 Table Participant characteristics, and number of selected electrodes by r^2^<0.05 criteria (from first fold only). (PDF)

S2 Table Correct classifications, when the timing of events is pre-designated. Sorted by stimulus type (note that each number is out of a possible 150 correct).
(PDF)

S3 Table Errors for Spontaneous Predictions. (PDF)

S1 Text Power spectral analysis. (PDF)

S2 Text Decoupling the cortical spectrum. (PDF)

S1 Fig Choice of collision time does not inform classifier about timing of events. Number of false predictions as a function of the choice of maximum distance between predicted event times (Collision time), for classification using both ERP and ERBB. The monotonic decay form and lack of “dips” or “peaks” shows that the collision time chosen did not inform the classifier about timing of stimuli. Of note, subject 5, who had the most early visual electrodes, was unaffected by even very low collusion times. The number of events correctly predicted was the same for every choice of collision time, so those data are not shown.
(TIF)
