# Supplementary material for: Spontaneous Decoding of the Timing and Content of Human Object Perception from Cortical Surface Recordings Reveals Complementary Information in the Event-Related Potential and Broadband Spectral Change
Source: PLoS Comput Biol. 2016 Jan 28;12(1):e1004660. doi: 10.1371/journal.pcbi.1004660 (PMC4731148; doi:10.1371/journal.pcbi.1004660)
Supplement: S1 Table — (PDF) [file pcbi.1004660.s001.pdf]

						ERP-responsive			ERBB-responsive		
subject	age	sex	Total	Rejected	Remaining	joint	face	house	joint	face	house
1	37	M	63	3	60	29	5	3	2	2	1
2	31	M	50	0	50	5	3	0	5	2	0
3	45	F	64	6	58	10	2	2	2	2	0
4	32	M	64	6	58	6	11	0	3	4	1
5		F	44	5	39	18	2	5	7	2	0
6	27	F	36	5	31	21	3	0	12	2	1
7	23	M	64	12	52	12	15	4	1	7	1
