# Supplementary material for: Spontaneous Decoding of the Timing and Content of Human Object Perception from Cortical Surface Recordings Reveals Complementary Information in the Event-Related Potential and Broadband Spectral Change
Source: PLoS Comput Biol. 2016 Jan 28;12(1):e1004660. doi: 10.1371/journal.pcbi.1004660 (PMC4731148; doi:10.1371/journal.pcbi.1004660)
Supplement: S2 Table — Sorted by stimulus type (note that each number is out of a possible 150 correct). (PDF) [file pcbi.1004660.s002.pdf]

| | Subject 1 | | Subject 2 | | Subject 3 | | Subject 4 | | Subject 5 | | Subject 6 | | Subject 7 | |
|---|---|---|---|---|---|---|---|---|---|---|---|---|---|---|
| | Faces | Houses | Faces | Houses | Faces | Houses | Faces | Houses | Faces | Houses | Faces | Houses | Faces | Houses |
| ERP | 127 | 144 | 128 | 145 | 85 | 145 | 137 | 149 | 145 | 142 | 145 | 144 | 107 | 146 |
| ERBB | 141 | 150 | 149 | 147 | 143 | 150 | 150 | 150 | 140 | 147 | 141 | 140 | 137 | 150 |
| Both | 139 | 147 | 150 | 150 | 124 | 149 | 150 | 150 | 145 | 148 | 147 | 144 | 137 | 150 |
