# Supplementary material for: Spontaneous Decoding of the Timing and Content of Human Object Perception from Cortical Surface Recordings Reveals Complementary Information in the Event-Related Potential and Broadband Spectral Change
Source: PLoS Comput Biol. 2016 Jan 28;12(1):e1004660. doi: 10.1371/journal.pcbi.1004660 (PMC4731148; doi:10.1371/journal.pcbi.1004660)
Supplement: S3 Table — (PDF) [file pcbi.1004660.s003.pdf]

	Number of captured stimuli			Number of false predictions, by time or class					Average absolute value of temporal error (for correct captures)		
	All	Face	House	All	Face - Time	Face - Class	House - Time	House - Class	All	Face	House
Subject 1											
ERP	268 / 300	141 / 150	127 / 150	33	4/23	23/23	1/10	9/10	19ms +/-14, (offset -2)	17ms +/-12, (offset 3)	22ms +/-16, (offset -7)
ERBB	266 / 300	130 / 150	136 / 150	55	3/3	0/3	37/52	21/52	32ms +/-33, (offset 10)	27ms +/-25, (offset 9)	37ms +/-38, (offset 11)
Both	281 / 300	144 / 150	137 / 150	16	0/10	10/10	0/6	6/6	18ms +/-13, (offset -1)	17ms +/-12, (offset 4)	19ms +/-13, (offset -6)
Subject 2											
ERP	278 / 300	135 / 150	143 / 150	35	6/8	6/8	12/27	18/27	25ms +/-26, (offset 10)	20ms +/-15, (offset 12)	29ms +/-32, (offset 7)
ERBB	291 / 300	144 / 150	147 / 150	8	2/2	1/2	3/6	3/6	35ms +/-30, (offset 18)	26ms +/-21, (offset 5)	44ms +/-35, (offset 31)
Both	290 / 300	145 / 150	145 / 150	12	4/4	4/4	6/8	2/8	19ms +/-18, (offset 9)	17ms +/-13, (offset 8)	22ms +/-21, (offset 11)
Subject 3											
ERP	241 / 300	143 / 150	98 / 150	74	15/60	56/60	11/14	5/14	20ms +/-16, (offset -2)	17ms +/-11, (offset -4)	24ms +/-20, (offset 2)
ERBB	275 / 300	142 / 150	133 / 150	34	3/3	0/3	31/31	4/31	30ms +/-26, (offset 4)	20ms +/-14, (offset 4)	39ms +/-32, (offset 3)
Both	285 / 300	144 / 150	141 / 150	23	7/15	12/15	5/8	5/8	19ms +/-15, (offset 1)	17ms +/-11, (offset -3)	22ms +/-17, (offset 5)
Subject 4											
ERP	290 / 300	150 / 150	140 / 150	18	1/10	10/10	8/8	1/8	18ms +/-15, (offset -2)	17ms +/-11, (offset -2)	20ms +/-19, (offset -2)
ERBB	276 / 300	143 / 150	133 / 150	35	4/9	9/9	21/26	7/26	38ms +/-35, (offset 1)	24ms +/-20, (offset 2)	53ms +/-41, (offset 1)
Both	295 / 300	150 / 150	145 / 150	8	1/2	2/2	6/6	0/6	19ms +/-17, (offset -4)	17ms +/-11, (offset -4)	21ms +/-21, (offset -4)
Subject 5											
ERP	293 / 300	148 / 150	145 / 150	7	0/5	5/5	0/2	2/2	18ms +/-12, (offset -7)	18ms +/-13, (offset -10)	18ms +/-11, (offset -5)
ERBB	283 / 300	139 / 150	144 / 150	17	0/6	6/6	0/11	11/11	25ms +/-19, (offset -2)	23ms +/-16, (offset 14)	27ms +/-21, (offset -18)
Both	291 / 300	150 / 150	141 / 150	8	0/8	8/8	0/0	0/0	18ms +/-12, (offset -7)	18ms +/-12, (offset -8)	18ms +/-12, (offset -7)
Subject 6											
ERP	286 / 300	142 / 150	144 / 150	18	4/10	7/10	3/8	5/8	20ms +/-15, (offset -7)	19ms +/-15, (offset -5)	20ms +/-15, (offset -9)
ERBB	266 / 300	139 / 150	127 / 150	35	0/21	21/21	3/14	11/14	27ms +/-20, (offset -5)	21ms +/-15, (offset 9)	34ms +/-22, (offset -21)
Both	290 / 300	147 / 150	143 / 150	10	0/7	7/7	0/3	3/3	21ms +/-17, (offset -5)	20ms +/-18, (offset 3)	22ms +/-16, (offset -14)
Subject 7											
ERP	266 / 300	143 / 150	123 / 150	52	11/30	28/30	17/22	10/22	22ms +/-22, (offset 1)	19ms +/-19, (offset 0)	26ms +/-25, (offset 2)
ERBB	281 / 300	135 / 150	146 / 150	17	4/4	1/4	3/13	11/13	34ms +/-34, (offset 21)	20ms +/-18, (offset 7)	48ms +/-39, (offset 34)
Both	291 / 300	142 / 150	149 / 150	10	1/1	0/1	2/9	7/9	21ms +/-19, (offset 4)	18ms +/-16, (offset 4)	24ms +/-22, (offset 5)
