# Supplementary material for: Spontaneous Decoding of the Timing and Content of Human Object Perception from Cortical Surface Recordings Reveals Complementary Information in the Event-Related Potential and Broadband Spectral Change
Source: PLoS Comput Biol. 2016 Jan 28;12(1):e1004660. doi: 10.1371/journal.pcbi.1004660 (PMC4731148; doi:10.1371/journal.pcbi.1004660)
Supplement: S1 Text — (PDF) [file pcbi.1004660.s004.pdf]

S1 Text - Power spectral analysis:

Power spectral analysis (for both power spectral snapshots and wavelet decomposition) was performed for frequencies between 1 and 200 Hz, in 1Hz bins, excepting line noise at 60Hz harmonics (57-63Hz, 117-123Hz, 177-183Hz).

Power spectral snapshots: A set of epochs surrounding the middle of each face or house visual stimulus and each inter-stimulus-interval (ISI) period, τ_q, were extracted from $V(t)$; each epoch was of duration T =1s, $\left(\tau_q - \frac{1}{2}T\right) < t < \left(\tau_q + \frac{1}{2}T\right)$. The power spectral density (PSD) of the epoch flanking time τ_q was calculated as

$$P(f,q) = \left| \frac{1}{\sqrt{T}} \sum_{t=-T/2}^{+T/2} V\left(\tau_q + t\right) H(t) e^{i2\pi f t} \right|^2$$

with Hann window (42)

$$H(t) = \frac{1}{2}\left(1 + \cos\left(\frac{2\pi t}{T}\right)\right).$$

Wavelet approach: A Morlet wavelet (43) of the form: $\psi(\tau,t) = \exp\frac{i2\pi t}{\tau}\exp\frac{-t^2}{2\tau^2}$ was convolved with the timeseries to get a time-frequency estimate for every $f = 1/\tau$:

$$\tilde{V}\left(1/\tau, t\right) = \sum_{t'=-5\tau/2}^{5\tau/2} V(t+t')\psi(t',\tau)$$

A total of 5 cycles (5τ) were used to estimate the amplitude and phase of the signal at each frequency for every point in time. In this way, a time-varying Fourier component $\tilde{V}\left(f,t\right) = r\left(f,t\right)e^{i\varphi(f,t)}$, with fixed uncertainty between the confidence in the estimate of the instantaneous amplitude and phase versus the confidence in temporal resolution is obtained at each frequency.
