# Supplementary material for: Spontaneous Decoding of the Timing and Content of Human Object Perception from Cortical Surface Recordings Reveals Complementary Information in the Event-Related Potential and Broadband Spectral Change
Source: PLoS Comput Biol. 2016 Jan 28;12(1):e1004660. doi: 10.1371/journal.pcbi.1004660 (PMC4731148; doi:10.1371/journal.pcbi.1004660)
Supplement: S2 Text — (PDF) [file pcbi.1004660.s005.pdf]

S2 Text - Decoupling the cortical spectrum:

The decoupling process to separate rhythmic activity from broadband change is described and illustrated in detail in [1], and illustrated specifically for this face-house context in [2]. It was applied here as follows:

Principal component decomposition of spectral change: The samples of the PSD, $P(f,q)$, (total N_q), were normalized prior to decomposition: $\widehat{P}(f,q)=\ln(P(f,q))-\ln\left(\frac{1}{N_q}\sum_q P(f,q)\right)$. A PCA method was used to determine the eigenvalues λ_j and eigenvectors $\vec{e}_j$ of the correlation matrix: $C(f,f')=\sum_q \widehat{P}(f,q)\widehat{P}(f',q)$. These eigenvectors, $\hat{C}\,\vec{e}_j=\lambda_j\,\vec{e}_j$, the ``Principal Spectral Components" (PSCs), reveal which frequencies vary together, and are ordered by magnitude of corresponding eigenvalue: $\lambda_1>\lambda_2>\cdots>\lambda_{N_f}$ ($N_f\equiv$ number of frequencies). If we define the rotation matrix $A(f,j)=(\vec{e}_1,\vec{e}_2,\cdots,\vec{e}_{N_f})$, then the projection, $W(j,q)$, of each individual original spectrum in the ensemble onto the new basis is $W(j,q)=\sum_f A(j,f)\widehat{P}(f,q)$.

The timecourse of broadband spectral change: The time-dependent, normalized, dynamic spectrum, $\widehat{P}(f,q)$, can be obtained in parallel fashion to the spectral snapshots. $P(f,t)=\frac{\left|\tilde{V}(f,t)\right|^2}{\frac{1}{N_t}\sum_t\left|\tilde{V}(f,t)\right|^2}$, and $\widehat{P}(f,t)=\ln(P(f,t))-\ln\left(\frac{1}{N_t}\sum_t P(f,t)\right)$. The reflection of the 1st PSC ($\vec{e}_1$) in the dynamic spectrum can be estimated by projecting the dynamic spectrum onto it: $\ln A(t)=\sum_f e_1(f)\widehat{P}(f,t)$. We call it $\ln A(t)$ here, because it approximates the logarithm of the time course of the coefficient of a power law in the cortical spectrum of the form $P(f,t)=A(t)f^{-\chi}$ [1]. The "broadband timecourse" for electrode n is obtained by smoothing $\ln A(t)$ with a Gaussian filter of standard deviation 80ms and then z-scoring (to put in intuitive units, because this measure is approximately normally distributed [3]), exponentiating, and subtracting 1 (so that it takes on a baseline value of 0): $B_n(t)=\exp\left(\ln A_n^Z(t)\right)-1$. The broadband power time course is meant to function as a time-varying estimate of changes in a multiplicative factor in the population firing rate [1,4].

1. Miller KJ, Sorensen LB, Ojemann JG, den Nijs M (2009) Power-law scaling in the brain surface electric potential. PLoS Comput Biol 5: e1000609.
2. Miller KJ, Honey CJ, Hermes D, Rao RP, denNijs M, et al. (2014) Broadband changes in the cortical surface potential track activation of functionally diverse neuronal populations. Neuroimage 85 Pt 2: 711-720.
3. Miller KJ, Hermes D, Honey CJ, Hebb AO, Ramsey NF, et al. (2012) Human motor cortical activity is selectively phase-entrained on underlying rhythms. PLoS computational biology 8: e1002655.
4. Miller KJ, Zanos S, Fetz EE, den Nijs M, Ojemann JG (2009) Decoupling the Cortical Power Spectrum Reveals Real-Time Representation of Individual Finger Movements in Humans. Journal of Neuroscience 29: 3132.
