# Supplementary figures and images for: Spontaneous Decoding of the Timing and Content of Human Object Perception from Cortical Surface Recordings Reveals Complementary Information in the Event-Related Potential and Broadband Spectral Change
Source: PLoS Comput Biol. 2016 Jan 28;12(1):e1004660. doi: 10.1371/journal.pcbi.1004660 (PMC4731148; doi:10.1371/journal.pcbi.1004660)

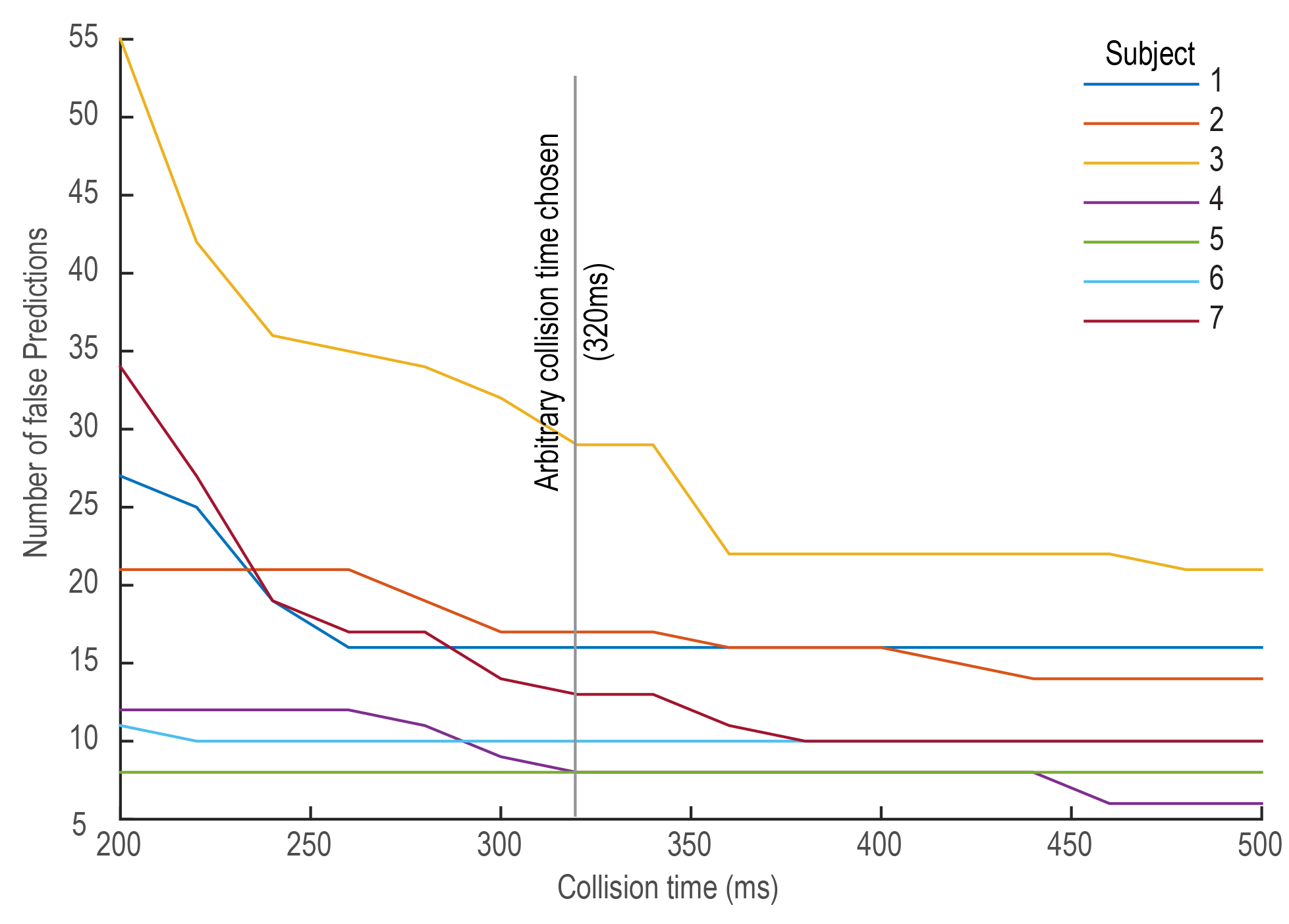

Supplement: S1 Fig — Number of false predictions as a function of the choice of maximum distance between predicted event times (Collision time), for classification using both ERP and ERBB. The monotonic decay form and lack of “dips” or “peaks” shows that the collision time chosen did not inform the classifier about timing of stimuli. Of note, subject 5, who had the most early visual electrodes, was unaffected by even very low collusion times. The number of events correctly predicted was the same for every choice of collision time, so those data are not shown. (TIF) [file pcbi.1004660.s006.tif]
